# Sepsis in Pregnancy: Recognition and Resuscitation

**DOI:** 10.5811/westjem.2019.6.43369

**Published:** 2019-08-06

**Authors:** Rachel E. Bridwell, Brandon M. Carius, Brit Long, Joshua J. Oliver, Gillian Schmitz

**Affiliations:** Brooke Army Medical Center, Department of Emergency Medicine, Fort Sam Houston, Texas

## Abstract

The normal physiologic changes of pregnancy complicate evaluation for sepsis and subsequent management. Previous sepsis studies have specifically excluded pregnant patients. This narrative review evaluates the presentation, scoring systems for risk stratification, diagnosis, and management of sepsis in pregnancy. Sepsis is potentially fatal, but literature for the evaluation and treatment of this condition in pregnancy is scarce. While the definition and considerations of sepsis have changed with large, randomized controlled trials, pregnancy has consistently been among the exclusion criteria. The two pregnancy-specific sepsis scoring systems, the modified obstetric early warning scoring system (MOEWS) and Sepsis in Obstetrics Score (SOS), present a number of limitations for application in the emergency department (ED) setting. Methods of generation and subsequently limited validation leave significant gaps in identification of septic pregnant patients. Management requires consideration of a variety of sources in the septic pregnant patient. The underlying physiologic nature of pregnancy also highlights the need to individualize resuscitation and critical care efforts in this unique patient population. Pregnant septic patients require specific considerations and treatment goals to provide optimal care for this particular population. Guidelines and scoring systems currently exist, but further studies are required.

## INTRODUCTION

In the United States, sepsis is the fourth leading cause of maternal death.^1^–^3^ Mortality in pregnant patients rose consistently at an average of 9% per year from 2001 to 2010 despite sepsis guidelines updates.^1^,^4^,^5^ As sepsis occurs in only 0.001% of pregnancies and in 0.002–0.01% of postpartum patients, data and consensus are limited regarding diagnostic and therapeutic interventions.^4^ Additionally, pregnancy is an exclusion criterion in all major sepsis trials to date, relinquishing clinical decisions to provider preference and expert opinion.^6^–^8^

## METHODS

In the following narrative review, we sought to comprehensively review the recent literature regarding sepsis in pregnancy. While pregnancy has been an exclusion criterion in every major sepsis trial as well as disease-specific trials, we identified all major observational trials, retrospective cohort studies for clinical rule derivation, and their subsequent validation studies.^6^–^8^ We also searched PubMed and Google Scholar from 1966 to October 2018 for English-language articles using a combination of keywords and medical subject headings “pregnancy” and “sepsis” for production of this narrative review, including case reports and series, retrospective and prospective studies, systematic reviews and meta-analyses, narrative reviews, and clinical guidelines. Three authors decided which studies to include for the review by consensus, with 122 resources selected for inclusion, focusing on ED evaluation and management. This review also highlights areas where more research is needed and underscores the protean nature of this complex physiology. As this is a narrative review and not a systematic review and/or meta-analysis, we did not grade the included resources or pool data.

## DISCUSSION

### The Pregnant Body: Shifting Homeostasis

The altered physiology of pregnancy can affect the immunologic response and clinical presentation of sepsis. Clinicians must be vigilant of the potentially competing priorities of mother and fetus, as physiologic changes brought on by sepsis in pregnancy in the mother can generate untoward effects on the fetus. It is essential to understand physiologic changes of normal pregnancy to appropriately approach sepsis in pregnancy. These changes occur secondary to altered hormonal levels that continue from conception to post-delivery, as well as anatomic transformations with fetal growth and uterine enlargement.^9^

Normal changes in pregnancy include a relative anemia due to expanding plasma volume that outpaces red blood cell growth.^10^ A baseline respiratory alkalosis develops from a rise in respiratory tidal volume with increased minute ventilation.^11^ Specific gastrointestinal differences likewise affect both normal baseline and disease resuscitation. The gravid uterus increases intragastric pressures, and high levels of progesterone and relaxin decrease lower esophageal sphincter tone.^12^,^13^ A normal delay in gastric emptying and elevation of the diaphragm up to four centimeters (cm) increase aspiration risk, elevating the risk of aspiration pneumonia and complicating intubating conditions.^14^

Throughout pregnancy the cardiovascular system undergoes a multitude of changes contributing to the physiology of mother and fetus. Systemic vasodilation begins early in the first trimester, decreasing systemic vascular resistance (SVR) by up to 35–40%, maintaining cardiac output due to a compensatory increase in heart rate.^15^ Late in the third trimester, heart rate peaks at rates up to 24% higher than the prepartum baseline.^15^,^16^ This translates to heart rate increases up to 30 beats per minute.^17^–^19^ Multiple gestations can further increase maternal heart rates.^20^ These compensatory cardiovascular changes generally return to baseline within two weeks of delivery, although a small proportion of patients maintain their pregnant cardiovascular measures at 12 weeks postpartum.^9^,^21^ Blood pressure may fall by 10–15 millimeters of mercury (mm Hg) in a normal pregnancy and nadir around 24 weeks gestation.^18^,^21^,^22^ Expanding plasma volume and red blood cell mass further work to offset lowered SVR and to maintain normotension from as early as six weeks into pregnancy until 34 weeks gestation.^19^

Pregnancy alone can increase white blood cell (WBC) counts to double pregestational levels.^23^ WBC counts may reach levels as high as 25,000 cubic millimeters (mm^3^) in a normal pregnancy.^24^–^27^ The physiologic stress of the peripartum period can push this leukocytosis further as high as 25,000/mm^3^ immediately postpartum.^24^ WBC counts may rise even higher in pre-eclampsia, complicating laboratory data interpretation.^28^,^29^ A clinical suspicion for pre-eclampsia taken with immunologic changes may cloud an infectious differential.^30^ Further normal physiologic changes in pregnancy are highlighted in Table 1.

Other pre-existing comorbidities may complicate physiologic alterations of pregnancy. Long-term medication use in pregnancy has increased commensurately with rates of obesity, non-insulin dependent diabetes mellitus, and hypertension.^31^,^32^ Prescription medication use during pregnancy has increased as much as 60% over the last 30–40 years.^33^ In the setting of infection, medications targeting blood pressure and glucose control can obscure physiologic responses. Non- or poorly-compliant pregnant patients further complicate this already-cloudy picture.

### The Evolution of Sepsis: Issues with Diagnosis and Guidance

The definition of sepsis continues to evolve. Previously, suspected infection source in conjunction with systemic inflammatory response syndrome (SIRS) criteria was key to identification^34^ Although these studies excluded pregnant patients, SIRS criteria nevertheless remained the primary standardized assessment tool for sepsis recognition.^34^ Before the second update to the sepsis guidelines in 2012, guidelines did not accurately identify maternal sepsis, identifying less than two-thirds of obstetric patients in retrospective reviews, highlighting the need to delineate pregnancy-specific guidelines.^3^,^35^

Working in parallel to the Surviving Sepsis Campaign, other parties presented criteria aimed at identifying maternal sepsis. The World Health Organization (WHO) modified the definition of maternal sepsis to “puerperal sepsis.”^36^ This narrow definition limited pregnant or postpartum sepsis to genitourinary tract infections between the time of rupture of membranes and six-weeks postpartum.^37^,^38^ The WHO provided a definition for septic abortion, which likewise remained isolated to genitourinary tract infections.^36^,^38^ As a result, many early maternal sepsis studies focused solely on the diagnosis and treatment of only these infections.^39^–^45^

Diagnoses were most recently supplemented in the Third International Consensus Definitions for sepsis and septic shock in 2016 by the Sepsis-related Organ Failure Assessment (SOFA) and the quick Sepsis-related Organ Failure Assessment (qSOFA) using SIRS criteria as fundamental principles.^46^,^47^ Similar to preceding trials, pregnancy was an exclusion criterion in these studies that established and validated the SOFA and qSOFA scores, thereby minimizing their utility in the pregnant population.^46^,^47^ As of this review, no studies have externally validating SOFA or qSOFA scores in pregnant patients, despite the fact that the components of these scores have been validated in various combinations in pregnant populations.^37^

The creation of two scores, the modified early warning scoring systems (MOEWS) and the sepsis in obstetrics (SOS) score, attempted to stratify pregnant patients with concern for sepsis; however, attempts to validate these scores have generated varying utility.^35^,^48^ MOEWS has a number of international variants (Table 2), limiting its application across regions and settings. MOEWS is generally hindered by its outcome “to help detect the early signs of illness and trigger timely medical review with appropriate intervention,” rather than specifically to target sepsis identification.^49^ The lone major MOEWS validation study analyzed 913 cases of chorioamnionitis, but only five cases met the definition of severe sepsis.^48^ Intended to predict severe sepsis by 2.0 guidelines, MOEWS restricts its utility not only by using a recently redefined term, but also by generating a myopic view of sepsis in pregnancy by focusing on chorioamnionitis and not the broader scope of sepsis sources.^49^

In 2014 the SOS sought to establish an obstetric-focused scoring system, incorporating the previously highlighted physiological changes in the cardiovascular, respiratory, and immune systems in pregnancy (Table 3).^50^ Based on the surviving sepsis campaign, the SIRS criteria overestimated morbidity and mortality in an obstetric cohort without accounting for normal physiologic changes.^51^–^55^ With this tailored scoring system, the authors sought to identify pregnant patients at high risk for sepsis with a primary outcome of intensive care unit (ICU) admission within 48 hours of admission. However, ICU admission criteria were not standardized.

Most recently, a single, prospective, internal validation trial of the SOS attempted to evaluate its performance with the same primary outcome.^56^ An SOS score of less than six points had a 64% sensitivity and 98.6% negative predictive value for excluding sepsis, although a score of six points or greater had a sensitivity of only 64% to diagnosis sepsis.^56^ Furthermore, of the 1250 pregnant patients presenting to the ED over a three-year study period, only 1.1% were admitted to the ICU, although ICU admission criteria remain unknown.^56^ While this lone, prospective validation study demonstrates a significant negative predictive value, additional validation studies and a larger sample population are needed to determine its utility in populations with different prevalence of septic pregnant patients.

Despite the need for obstetric-focused scoring systems in sepsis, emergency providers lack substantially validated criteria or schema to bolster decision-making and hospital admission when confronted with a sick pregnant or postpartum patient.

### Treatment Considerations Specific to Pregnancy

#### Pneumonia

Pneumonia is responsible for 30% of infections in pregnant patients with severe sepsis, carrying significant morbidity for both mother and fetus.^5^ In one study, up to one-fifth of pregnant patients experienced a delay in pneumonia diagnosis, while half experienced significant morbidities such as empyema and respiratory failure.^57^ Initial diagnosis is often made by chest radiograph. Appropriate shielding of the abdomen exposes the fetus to less than 0.01 milliGray (mGy), well below the threshold of adverse effects.^58^ The lung may be upwardly displaced by the growing uterus, and the increased density of parenchyma can make definitive diagnosis difficult.^59^ Ultrasound (US) has a 94–97% sensitivity and 94–96% specificity for pneumonia diagnosis in a recent meta-analysis.^59^,^60^ Although chest computed tomography (CT) is rarely required, it can be safely performed if needed for diagnosis.^46^

The most common microbial causes of pneumonia in pregnancy include *S. pneumoniae* and *H. influenzae*.^61^ Antibiotic coverage should treat these pathogens. However, other sources to consider include *Legionella* spp., Varicella zoster, and *Pneumocystis jirovecii* in patients with human immunodeficiency virus (HIV).^13^ While fluoroquinolones should be avoided, penicillins, cephalosporins, and macrolides are all considered safe to use in pregnancy.^62^ For pregnant patients admitted to the ICU, both *S. pneumoniae* and *Legionella* spp. should be covered.^62^ A pneumococcal beta lactam, such as cefotaxime or, if not peripartum, ceftriaxone, and a macrolide should be administered.^13^,^62^ Vancomycin and linezolid do not currently have established safety in pregnancy, but should be considered in cases where methicillin-resistant *Staphylococcus aureus* is suspected.

In a small case series, 59% of pregnant patients with pneumocystis pneumonia required mechanical ventilation due to respiratory failure.^63^ The authors found a 50% mortality rate for the mothers and 41% mortality for combined fetus and neonates.^63^ These patients should be treated similarly to their non-pregnant counterparts with trimethoprim-sulfamethoxazole and corticosteroids if the A-a gradient is greater than 35 or the partial pressure of oxygen (PaO_2_) is less than 70 mm Hg.^64^ The mother should also be monitored for immune reconstitution inflammatory syndrome.^64^ If treatment is active at the time of delivery, the neonate should be monitored for hyperbilirubinemia.^64^

The course of pneumonia in pregnant patients can be further complicated by decreased secretion clearance and worsening airway obstruction.^13^,^61^ Secondary to pregnancy physiology and treatments routinely administered in the course of delivery, aspiration during labor represents another significant source of infection.^14^ Epidural blocks may blunt or inhibit the cough reflex, further increasing the risk of aspiration pneumonitis and pneumonia.^65^

Pregnancy was an exclusion criteria in the PROTECT (prophylaxis for thromboembolism in critical care) trial, which examined risk factors for mortality secondary to pneumonia in patients admitted to the ICU.^66^ ICU admission threshold should be lower for pregnant individuals, as they have decreased tolerance for hypoxemia and may quickly deteriorate with pneumonia.^13^ Blood gas interpretation in pregnant patients should take into account the expected physiologic alkalemia, which may blunt initial laboratory findings of hypercapneic respiratory failure.^14^ Anatomical compression of the inferior vena cava in late pregnancy can reduce cardiac preload causing hypotension, exacerbated by the addition of positive pressure from mechanical ventilation.^11^,^24^ This may necessitate placing the patient in the left lateral decubitus position.^67^ Prevention of maternal hypoxemia is critical, as this quickly leads to fetal decompensation. Thus, maintaining a PaO_2_ greater than 70 mm Hg can prevent deleterious effects on the fetus.^68^,^69^ Although extrapolated from asthma data and therefore controversial, partial pressure of carbon dioxide should be maintained between 28–32 mm Hg to prevent fetal acidemia and maternal hypercapnia.^69^

#### Pyelonephritis

Pyelonephritis in pregnancy is a complicated infection requiring intravenous (IV) antibiotics and admission for continued monitoring of mother and fetus.^70^,^71^ Pyelonephritis occurs in approximately 2% of pregnancies in the U.S. but accounts for the largest proportion of maternal inpatient admissions.^71^ Up to 20% of cases occur in the second and third trimester.^70^–^72^ Numerous factors predispose pregnant women to pyelonephritis: dilation of the renal calyces secondary to progesterone; stagnation of ureteral peristalsis; mechanical compression of the bladder; and increased glomerular filtration rate, resulting in glucosuria and alkaluria facilitating bacterial growth.^73^

Acute pyelonephritis in pregnancy can significantly increase the risk of maternal anemia, acute renal failure, respiratory distress, and preterm birth.^4^ Additionally, patients with maternal pyelonephritis demonstrate a 33% increased risk of chorioamnionitis, further predisposing them to sepsis.^74^ More than 80% of acute pyelonephritis cases in pregnancy are from *E coli*, but other uropathogens include *Klebsiella, Streptococcal* spp*., Proteus mirabilis*, and *Enterococcus*.^74^,^75^ Although pregnant patients are specifically excluded from Infectious Diseases Society of America (IDSA) guidelines, ceftriaxone, cefepime, or ampicillin plus gentamicin are feasible treatment options.^74^,^75^ In patients less than 24 weeks gestation, intramuscular ceftriaxone has demonstrated equal efficacy in length of hospitalization and days until resolution of infection compared to IV ampicillin and gentamicin or cefazolin.^76^ Ceftriaxone should be avoided in the periparturition period, however, due to the risk of neonatal kernicterus.^67^ Urine culture and local resistance patterns should guide empiric therapy.^73^

Carbapenems or piperacillin-tazobactam could be considered for broader coverage in immunocompromised patients or those with severe pyelonephritis impairing urinary drainage; however, imipenem should be avoided due to adverse fetal effects demonstrated in vivo.^70^,^73^ *E. coli* and other gram-negative rods cause the vast majority of pyelonephritis in pregnancy, carrying the potential for large-scale endothelial cell damage in capillary beds from endotoxin release.^73^–^75^ This endothelial damage commonly affects renal and pulmonary tissue, resulting in acute respiratory distress syndrome in 1–8% of cases, further complicating the maternal patient.^73^ Unlike the non-pregnant population, a test of cure is required in maternal patients following clinical resolution.^67^

#### Appendicitis

Appendicitis occurs less frequently in pregnancy (approximately 1 in 1500) and peaks in the second trimester compared to the non-pregnant population.^77^–^79^ However, 1 in 1000 pregnancies undergo surgical evaluation for possible appendicitis, with increased rates of surgical intervention due to increased perforation risk as well as mortality.^77^,^78^ Maternal mortality secondary to appendicitis is 4%, and complications of perforated appendicitis result in an estimated 43% fetal mortality rate.^80^,^81^

Physiologic changes of pregnancy and atypical presentation make maternal diagnosis particularly challenging. The fundus rises and displaces the appendix from the right lower quadrant (RLQ).^81^ Fundal displacement of the omentum prevents it from sealing off an inflamed appendix.^82^ RLQ pain and tenderness are the presenting symptoms in 75% of maternal appendicitis, while another 20% of cases present with right upper quadrant pain.^83^ However, up to 45% of these cases present with rectal tenderness, which is not commonly associated with or examined with suspected appendicitis.^83^ Nausea and vomiting, common in pregnancy, can further complicate the clinical picture. Therefore clinicians should note any significant changes to the patient’s “normal” course of “morning sickness” during the history. Maternal leukocytosis is not reliable for diagnosing appendicitis or perforation due to normal physiologic changes. However, the presence of bilirubinemia greater than 1.0 milligrams per deciliter (mg/dL) has demonstrated sensitivity of 70% and specificity of 86% in evaluating for perforation in appendicitis, which may aid clinical judgment.^84^

While ultrasound (US) is safe in pregnancy, wide variation in appendiceal location makes evaluation difficult. Sensitivity and specificity of US for the diagnosis of maternal appendicitis ranges from 67–100% and 83–95%, respectively.^85^ The lower range is significantly less compared to non-pregnant populations in ED-performed bedside US, where sensitivity and specificity approximate 49.5–86.2% and 91.4–99.7%, respectively.^86^ In cases where US is equivocal, magnetic resonance imaging (MRI) is recommended, sparing ionizing radiation to both mother and fetus.^87^ A meta-analysis of MRI in the diagnosis of maternal appendicitis demonstrated a sensitivity of 96.8% and a specificity of 99.2%.^88^

MRIs are routinely run without gadolinium, which poses no hypothetical risk to the fetus.^87^ Early antibiotic coverage should be initiated with a second-generation cephalosporin and clindamycin or metronidazole.^89^ Prompt surgical consultation should be obtained, as the risk of perforation rises with delaying surgical involvement for more than 24 hours.^89^,^90^ Additionally, the risk of fetal loss increases with perforation of the appendix, with a 36% rate of fetal loss, compared to 1.5% without appendiceal rupture, underscoring the importance of early surgical consult in conjunction with antibiotics.^91^

#### Pelvic Inflammatory Disease

Although rare, maternal sepsis from pelvic inflammatory disease (PID) is associated with high-mortality for mother and fetus, as well as increased risk of preterm delivery.^92^ PID in pregnancy presents typically in the first trimester with fever and abdominal pain, adnexal tenderness, and cervical motion tenderness. Bacteria can ascend prior to the mucus plug sealing off the decidua around 12 weeks.^93^ PID may rapidly progress to tubo-ovarian abscess (TOA), with a mortality up to 9%.^94^ TOA presents similarly to an infected ectopic pregnancy with fever and adnexal tenderness. The presentation of fever, leukocytosis, and diarrhea should prompt consideration of TOA, independently predicted by elevated C-reactive protein.^95^ Pregnancy with PID requires hospitalization for treatment.^92^ Doxycycline, the mainstay treatment for PID per IDSA guidelines, has been repeatedly proven to have severe teratogenicity and therefore should not be used.^92^ Azithromycin should be substituted, in conjunction with an IV second-generation cephalosporin such as cefotetan or cefoxitin.^92^ Penicillin cross-reactivity with second-generation cephalosporins is negligible, providing effective treatment in pencillin-allergic patients.^96^,^97^ This regimen also covers *Mycoplasma genitalium*, which accounts for up to 8.7% of non-chlamydial and non-gonococcal PID cases.^98^

#### Endometritis

Endometritis presents with postpartum fever, tachycardia, and foul lochia or malodorous vaginal discharge and occurs with ascension of bacteria during labor that colonizes amniotic fluid and decidua.^67^ Cases are generally polymicrobial, with two-thirds containing both anaerobic (Bacteroides, Clostridium, and Peptostreptococcus spp.) and aerobic bacteria (Group B Streptococcus, E. coli, and enterococcus).^99^ The presence of a hematoma is concerning for S. pyogenes and S. aureus and toxic shock syndrome.^77^ IV gentamicin and clindamycin are efficacious, although this regimen does not cover enterococcus.^100^ Doxycycline plus cefoxitin or ampicillin/sulbactam is an additional regimen. In those who do not respond within the first 48–72 hours, ampicillin is added to cover for these pathogens.^101^ In patients delivering via cesarean section (C-section) who develop endometritis, parametrial cellulitis with phlegmon formation in the broad ligament or, less-commonly, parametrial phlegmon can cause persistent fevers and require interventional radiology consult for drainage.^101^ Venous drainage post-C-section can also spread infection, generating septic pelvic thrombophlebitis.^102^

Pelvic thrombophlebitis is usually refractory to broad-spectrum antibiotics alone and requires anticoagulation with broad polymicrobial coverage.^102^–^104^ Liberal use of postpartum CT has significantly impacted management. In a retrospective cohort study of 238 postpartum patients, the use of CT resulted in alteration of antibiotic therapy in 10%, addition of low-molecular weight heparin (LMWH) in 12%, and surgical intervention in 17%.^105^ This study demonstrated that the addition of CT significantly impacted the clinical course of approximately 40% of patients.^105^ Table 4 summarizes these infections.

### Approach to Resuscitation in Pregnancy

Optimal stabilization of the fetus depends on adequate resuscitation of the mother.^77^ Initial resuscitation should include IV fluid administration and optimized positioning. The left lateral decubitus position maximizes patient hemodynamics in the third trimester, improving preload by decreasing inferior vena cava compression.^77^ Fluid resuscitation should begin within the first three hours of presentation with an initial recommended volume of 30 milliters per kilogram of crystalloid if either hypotension or lactic acid >4 millimoles per liter (mmol/L) is present.^107^ Due to increased blood volume in pregnancy, a lactic acid threshold of 4 mmol/L may lack sensitivity in this population. In a retrospective cohort of 159 septic pregnant patients, the mean lactic acid level of those admitted for ICU level care was 2.6 mmol/L, and those with positive blood cultures had a level of 2.2 mmol/L.^108^ This study found increased morbidity with elevated lactic acid, with an adjusted odds ratio of 2.34 per 1 mmol/L increase in lactic acid.^108^

No specific guidelines exist for vasopressors preference in pregnant patients. Although there is no explicit recommendation for mean arterial pressure optimization for sepsis in pregnancy, 65 mm Hg is a reasonable resuscitation goal.^107^ Fetal monitoring can provide further titration feedback.^109^ The 2016 Society of Critical Care Medicine guidelines do not offer recommendations tailored for the pregnant patient, although their current data support the use of norepinephrine as the first-line vasopressor in pregnant septic patients.^77^,^107^,^110^ Due to the paucity of data, there is scant evidence to suggest that administration of norepinephrine causes negative fetal outcomes, or to suggest how norepinephrine administration impacts fetal outcome.^111^

The choice for second-line vasopressor has been extrapolated from controlled studies with spinal anesthetics and is therefore controversial for application in sepsis.^112^–^116^ Phenylephrine and ephedrine are often used as second-line agents, although with known tachyphylaxis.^110^^7^,^114^,^116^ Unlike ephedrine, phenylephrine does not alter the fetal acid-base status, although its alpha stimulation generates reflex maternal bradycardia and diminished cardiac output.^114^,^115^ In comparison, ephedrine does not generate bradycardia, although its indirect action to release pre-existing maternal catecholamines may prove less efficacious in a septic patient who has already exhausted her endogenous stores and expended her cardiac reserve.^113^–^115^

The data on vasopressor use in pregnancy are typically derived from C-section deliveries, many of which are elective.^65^,^113^,^114^ In the Task Force on Obstetric Anesthesia, the American Society of Anesthesiologists recommended phenylephrine over ephedrine because of the preferred fetal acid-base status, as ephedrine causes fetal acidemia.^112^ While this choice was supported by an international consensus of counterpart agencies, these data are extrapolated from a different physiologic context.^117^

In the rare setting of septic myocarditis, dobutamine is the preferred inotrope.^113^ Despite its very limited use in the non-pregnant septic population, dobutamine presents a viable option to improve inotropy in pregnant patients already on vasopressors and fluids.^112^,^118^ Based on previous ovine models, dobutamine provides inotropy in pregnant sheep, although it decreases uterine blood flow; it requires further study in humans.^119^

Other treatment considerations in maternal sepsis include glucose control, steroids, and venous thromboembolism (VTE) prophylaxis. Maternal hyperglycemia can directly cause fetal hyperglycemia and ultimately acidosis, decreasing uterine blood flow and lowering fetal oxygenation.^109^ Maternal blood glucose should be maintained less than 180 grams per deciliter.^109^ Steroids are recommended by the American College of Obstetrics and Gynecology in women between 24 weeks and 33 weeks and six days who are at risk of a preterm delivery within seven days, which is inclusive of those with rupture of membranes.^120^ Hydrocortisone should be considered in those patients who do not improve with IV fluids and vasopressors.^107^,^110^ Pregnancy alone confers a five-fold increased risk of deep vein thrombosis as compared to the non-pregnant population.^122^ In individuals in septic shock on VTE prophylaxis, there was a 37% incidence in VTEs despite these prophylactic interventions. As septic pregnant patients are at high risk of VTE, patients without contraindications should receive both intermittent compression devices and either daily LMWH or 2–3 times daily administration of unfractionated heparin.^109^,^122^ Direct oral anticoagulants are not currently recommended.^109^

## CONCLUSION

The anatomic and physiologic changes of pregnancy pose a challenge in early recognition and management of sepsis. Current sepsis guidelines were extrapolated from randomized control trials that specifically excluded pregnant patients. Although new guidelines have been created to risk stratify pregnant patients, they are without significant validation. Further research and validation are needed to help properly recognize and treat this small but critically ill population to improve outcomes for both mother and fetus.

## Figures and Tables

**Table 1:** Physiologic changes during pregnancy.^4^

System	Baseline Changes	Physiologic Impact
Cardiovascular	Decreased arterial pressure Increased heart rate and cardiac output	Increased risk of hypoperfusion in sepsis Abnormal baseline may mask signs of sepsis
Gastrointestinal	Decreased esophageal tone and delayed gastric emptying	Aspiration pneumonia risk Increased aspiration risk with airway interventions
Genitourinary	Decreased vaginal pH	Increased risk of chorioamnionitis
Hematology	Increased plasma volume without proportional increase in red cell mass, hemoglobin Increased production of factors VII, VIII, IX, X, XII and von Willebrand factor	Physiologic anemia, decreased O_2_ supply to tissues Increased risk of disseminated intravascular coagulation and venous thromboembolic disease
Respiratory	Increased tidal volume and minute ventilation with typically unchanged respiratory rate Decreased residual volume due to elevated diaphragm	Decreased PaCO_2_ levels (A“normal” blood gas may therefore reflect impending respiratory failure.) Decreased oxygenation with faster rate of desaturation
Renal	Ureteral dilation and increased vesicoureteral reflux Increased renal plasma flow and glomerular filtration rate	Increased risk of pyelonephritis Abnormal baseline may mask renal injury in sepsis

*PaCO_2_*, partial pressure of carbon dioxide.

**Table 2:** Versions of the modified obstetric early warning scoring systems (aggregate score MOEWS).^35^

Variable	Low abnormal range			Normal	High abnormal range			
Score	3	2	1	0	1	2	3	Trigger
Heart rate	≤39	40–59	60–74	75–104	105–109	110–129	≥130	
Systolic blood pressure	≤79		80–89	90–139	140–149	150–199	≥200	Medium Risk: Score 4–5
Respiratory rate	≤5	5–9	10–14	15–19	20–24	25–29	≥30	
Temperature	≤34.9		35–35.9	36.0–37.9	38.0–38.4		≥38.5	High Risk: Score^3^ 6
Oxygen saturation	≤87	88–89	90–94	95–100				
Mental status				Alert	Voice	Pain	Unresponsive	

**Table 3:** The Sepsis in Obstetrics Score (SOS) scoring criteria.^58^

Variable	Low abnormal range				Normal	High abnormal range			
Score	4	3	2	1	0	1	2	3	4
Heart rate					≤119	120–129	130–149	150–179	≥179
Systolic blood pressure	<70		70–90		>90				
Respiratory rate	≤5		6–9	10–11	12–24	25–34		35–49	>49
Temperature		≤34.9		35–35.9	36.0–37.9	38–38.4		≥38.5	High Risk Score ≥ 6
Oxygen saturation	≤85%	85–89%		90–91%	≥92%				
White blood cell count	<1		1–2.9	3–5.6	5.7–16.9	17–24.9	25–39.9		>39.9
% Bands					<10%		≥10%		
Lactic acid					<4		≥4		

**Table 4:** Chronologic presentation of sepsis etiologies and recommended antibiotics.

Infection	Time Frame	Evaluation	Management
Pelvic inflammatory disease	1st trimester	Pelvic examination, transvaginal ultrasound to evaluate for tubo-ovarian abscess if suspected^93^–^95^	Azithromycin and cefoxitin^92^
Appendicitis	2nd trimester more commonly than 1st and 3rd trimester	Ultrasound, if equivocal then magnetic resonance imaging	Definitive treatment is surgery, cefoxitin + clindamycin, cefoxitin + metronidazole^89^
Pyelonephritis	2nd and 3rd trimester more commonly than 1st trimester	Urinalysis, urine culture; obtain imaging to evaluate for renal abscess if patient is clinically toxic or hemodynamically unstable^70^,^71^,^73^	Immunocompetent: ceftriaxone, cefepime, ampicillin + gentamicin Immunocompromised: piperacillin/tazobactam, carbapenem^73^,^75^,^76^,^106^
Pneumonia	1st, 2nd, and 3rd trimester	Chest radiograph, consider ultrasound^46^,^58^–^60^	Pneumococcal beta-lactam + macrolide MRSA coverage if suspected: vancomycin, linezolid^12^,^62^
Endometritis	Post-partum	Computed tomography^105^	IV gentamicin + clindamycin, doxycycline + cefoxitin, ampicillin/sulbactam^100^,^101^

*MRSA*, methicillin-resistant *Staphylococcus aureus*; *IV*, intravenous.
